# Performance of Contactless Respiratory Rate Monitoring by Albus Home^TM^, an Automated System for Nocturnal Monitoring at Home: A Validation Study

**DOI:** 10.3390/s22197142

**Published:** 2022-09-21

**Authors:** William Do, Richard Russell, Christopher Wheeler, Megan Lockwood, Maarten De Vos, Ian Pavord, Mona Bafadhel

**Affiliations:** 1 Albus Health, Oxford OX4 2HN, UK; 2 Respiratory Medicine Unit, Nuffield Department of Medicine, University of Oxford, Oxford OX3 7FZ, UK; 3 Royal Brompton Hospital, London SW3 6NP, UK; 4 Department of Electrical Engineering and Department of Development and Regeneration, KU Leuven, 3000 Leuven, Belgium; 5 King’s Centre for Lung Health, School of Immunology and Microbial Sciences, Faculty of Life Sciences and Medicine, King’s College London, London SE1 1UL, UK

**Keywords:** validation, nocturnal, monitoring, respiratory rate, breathing, contactless, automated, remote, trials

## Abstract

Respiratory rate (RR) is a clinically important predictor of cardio-respiratory deteriorations. The mainstay of clinical measurement comprises the manual counting of chest movements, which is variable between clinicians and limited to sporadic readings. Emerging solutions are limited by poor adherence and acceptability or are not clinically validated. Albus Home^TM^ is a contactless and automated bedside system for nocturnal respiratory monitoring that overcomes these limitations. This study aimed to validate the accuracy of Albus Home compared to gold standards in real-world sleeping environments. Participants undertook overnight monitoring simultaneously using Albus Home and gold-standard polygraphy with thoraco-abdominal respiratory effort belts (SomnomedicsEU). Reference RR readings were obtained by clinician-count of polygraphy data. For both the Albus system and reference, RRs were measured in 30-s segments, reported as breaths/minute, and compared. Accuracy was defined as the percentage of RRs from the Albus system within ±2 breaths/minute of reference counts. Across a diverse validation set of 32 participants, the mean accuracy exceeded 98% and was maintained across different participant characteristics. In a Bland–Altman analysis, Albus RRs had strong agreement with reference mean differences and the limits of agreement of −0.4 and ±1.2 breaths/minute, respectively. Albus Home is a contactless yet accurate system for automated respiratory monitoring. Validated against gold –standard methods, it enables long-term, reliable nocturnal monitoring without patient burden.

## 1. Introduction

Respiratory rate (RR) is an essential clinical measurement when assessing states of health and disease and is a known predictor of cardio-respiratory deteriorations in hospital and home environments [1,2,3]. Similarly, dyspnoea is an important symptom, but is often poorly captured, being dependent on subjective memory or patient-reported outcome measures [4,5]. Despite the clinical utility of RR, few reliable monitoring methods are available. For the majority of patients, RR is measured by visual observation and counting, shown to be highly variable even between healthcare professionals by up to 6 breaths per minute [6]. Moreover, manual RR counts are time-consuming and limited to few discrete readings.

The gold standard for continuous overnight RR monitoring consists of a manual count of nocturnal polygraphy data captured through several burdensome sensors, such as nasal cannula for airflow or thoraco-abdominal respiratory effort belts [7,8]. These devices are uncomfortable for patients, dislodged easily, and are not suitable for more than one or two days of use. Moreover, manual scoring of polygraphy data is highly time intensive. Due to these limitations, there has been increasing interest in more practicable solutions for RR monitoring for night-time and at home. A common approach has been wearable devices, which range from body-worn ‘smart clothing’ to devices and patches attached to skin using adhesives [9,10,11]. However, though less burdensome than polygraphy, wearables still suffer from the need for active adherence, regular charging, and inconvenience, which lead to significant decreases in adoption after initial periods of novelty [12]. There have also been minimally- and fully-contactless approaches. For instance, through ballistocardiography signal processing, bed sensors can derive respiratory rates [13]. However, as they need to be placed under mattresses, a variety of factors can deteriorate the signal in real-life use, such as differing mattress type and thickness. Camera-based solutions can also capture vital signs [7], but are limited by privacy and acceptability concerns, and primarily targeted for high-risk environments, such as mental health hospitals and prisons [14].

Addressing the need for a fully contactless, automated, and minimally-intrusive solution, here we present Albus Home^TM^, a CE-marked bedside system integrating a radiofrequency radar-based respiratory monitoring system together with acoustic and environmental sensors for monitoring multiple clinical metrics in one device. Previous validation studies of contactless RR monitoring solutions have been performed in highly-controlled laboratory environments or sleep study settings [7,8]. However, these are environments without the significant natural variability that exists in home sleeping conditions, such as different beds, duvets, clothing, sleeping habits and positions, and the presence of bed-sharers. The aim of this work was to evaluate the performance and accuracy of the RR monitoring component of the Albus system against reference readings taken from polygraphy in a real-world context, where participants were monitored in their usual bedrooms’ sleeping conditions.

## 2. Materials and Methods

### 2.1. Albus System: Albus Home^TM^ Monitoring Device and RR Analytics

**Figure 1:**
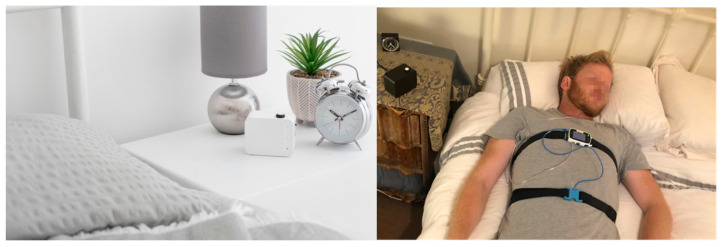
Image showing the Albus Home at the bedside (**left**) and the reference polygraphy device using thoraco-abdominal respiratory effort bands (**right**). For illustration, duvets were removed to show the device, but participants slept using own duvets under usual sleeping conditions.

Albus Home RD (Research Device) is a compact contactless and automated bedside monitoring device that functions by plug-and-play and works automatically after being placed at the bedside (Figure 1). To capture RR without the need to do or wear anything, the system uses a radiofrequency doppler radar-based motion sensor that detects the fine movements of the chest and abdomen that occurs during respiration. Overnight recordings from the Albus Home RD are then analysed using a proprietary and automated Albus RR signal processing system (v1), which reports RR as breaths per minute.

### 2.2. Participants

All participants included in this validation took part in a UK Health Research Authority Research Ethics Committee (20/YH/0041) approved study and provided written informed consent. Participants consisted of adults and children, including healthy volunteers and those with stable underlying respiratory conditions. Participants who required night-time ventilation aids and devices at home were excluded. All participants were monitored in their usual home bedroom environments, with nocturnal recording hours set to encompass their usual sleeping schedules. Participants were either provided with the Albus Home RD at in-person visits or received them by post after a virtual visit. Participants set up the device themselves at home after watching a brief setup video or through a remote video-call, during which a clinician also confirmed the correct application of the polygraphy device. Instructions provided to the participants comprised a brief setup guide for the placement of the Albus device at the bedside, but otherwise participants were reassured they could sleep in their usual ways, including positions, bedding, and clothing.

### 2.3. Validation and Analysis

In addition to the Albus Home RD^TM^, participants were simultaneously monitored overnight using an AASM- (American Academy of Sleep Medicine, Darien, IL, USA) and FDA (U.S. Food and Drug Administration, Silver Spring, MD, USA)-approved nocturnal polygraphy device with thoraco-abdominal respiratory effort belts (Somnotouch Respiratory^TM^, Somnomedics EU, Randersacker, Germany). Gold-standard reference RR readings were obtained by a clinician counting the number of respiratory cycles in each 30-s segment from raw respiratory effort traces from polygraphy (Dominolight^TM^ software, Somnomedics EU). For both the Albus system and reference, RR readings were measured in 30-s segments and reported as breaths per minute. This validation dataset was fully held-out, meaning that these data were not used during the development of the algorithms.

Accuracy was analysed and reported for periods starting from when each participant was in bed and there were ten minutes of respiratory data without gross motion from both the Albus system and polygraphy. As per precedent validation methodology [15], a 15-minute period was counted and compared for each participant to assess the inter- and intrasubject performance. A RR reading from the Albus system was defined as accurate when within ±2 breaths per minute of the reference count as per criteria used in previous work [8,16]. The % accuracy was calculated per participant, which was defined as the proportion of accurate RR readings over all RR readings. Additionally, the correlation and agreement in RR readings between the Albus system and reference were evaluated through a Pearson’s correlation coefficient and Bland–Altman analysis, respectively [10,16]. Finally, to assess the performance of the system in longer periods of continuous monitoring and for providing hourly mean RR results, a more substantive annotation and comparison was performed, which comprised the first 2 h for the first 10 consecutive participants. Participants performed full overnight recordings using the Albus system and reference, and the Albus system output RR readings throughout the night. However, the lengths of time compared for this analysis was limited by the extensive time and resources needed to obtain manual clinician-counts of RR from the polygraphy (in contrast to automated RR output from the Albus system). Individual 30-s segments where a manual count was not possible from the reference polygraphy were excluded from the analysis. In order to assess the reliability of the Albus system in addition to its accuracy, the proportion of segments where a RR was available from a manual count but not Albus system was quantified and reported.

## 3. Results

A total of 32 adult and pediatric participants underwent overnight recording with the Albus Home RD and polygraphy in parallel in their usual sleeping conditions at home. Eighteen participants were healthy volunteers (15 adults, 3 children), whilst 14 had an underlying chronic respiratory condition (9 adults, 5 children; 10 asthma, 3 chronic obstructive pulmonary disorder, 1 pulmonary sarcoidosis). The summary characteristics of the validation set participants are shown in Table 1.

Across 480 min (15-min periods for 32 participants), individual RR readings (over 30-s segments) were compared between the Albus system and reference manual counts. A polygraphy RR count was not available from 24.5 min and was excluded from analysis. Of the remaining periods, a RR was not available from the Albus system from 5.5 min and was excluded. Thus, RR readings were compared between Albus and reference for the remaining 450 min (900 RR readings)—mean accuracy (SD) was 99.0% (1.9) and mean absolute error (SD) was 0.6 (0.1) breaths per minute.

**Figure 2:**
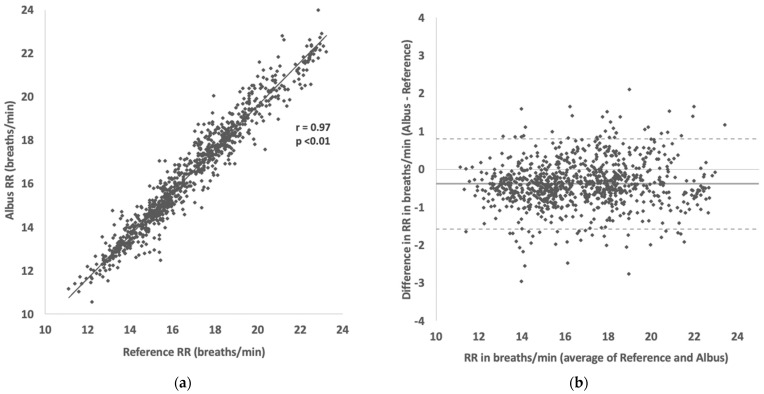
(**a**) Scatter plot showing correlation between RR readings between Albus system and reference (clinician-counted polygraphy). (**b**) Bland–Altman plot showing agreement in RR readings between Albus system and reference (solid line shows mean difference of −0.38 breaths/min and dotted lines show 95% limits of agreement from 0.8 to −1.6 breaths/min). There are, in total, 900 comparisons across 32 participants, with each point depicting a RR reading for a 30-s period.

Accuracy was maintained across participants with different characteristics: 98.6% (2.2) for healthy vs. 99.5% (1.3) for those with respiratory conditions; 99.0% (2.0) for adults vs 99.0% (1.8) for children; and 99.3% (1.8) for single-sleepers vs 98.2% (1.9) for bed/room-sharing participants. There was a strong correlation of RR readings between the Albus system and reference counts (r = 0.97, *p* < 0.01, Figure 2A), and close agreement in the Bland–Altman analysis (Figure 2B) with 95% limits of agreement (1.96 × SD) at ±1.2 breaths per minute. The Albus system reported marginally lower RRs compared to reference with a mean difference of −0.4 breaths per minute (deemed non-significant as less than half a breath).

**Figure 3:**
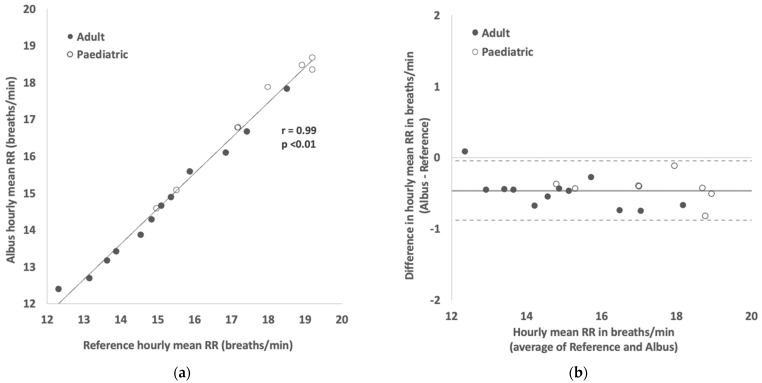
(**a**) Scatter plot showing correlation between hourly mean RR readings between Albus system and reference (clinician-counted polygraphy). (**b**) Bland–Altman plot showing agreement in hourly mean RR readings between Albus system and reference (solid line shows mean difference of −0.46 breaths/min and dotted lines show 95% limits of agreement from 0.04 to −0.88 breaths/min). There are, in total, 20 comparisons across 10 participants, with each point depicting a mean RR reading for a 1-h period.

In the subset of 10 participants (first 10 consecutively recruited; 6 adults aged 25–50 years and 4 children aged 6–12 years) where a further 2 h of polygraphy reference data were manually counted, hourly mean RRs were compared between the Albus system and reference, again showing a strong correlation (r = 0.99, *p* < 0.01, Figure 3A) and close agreement (Figure 3B) with mean difference and limits of agreement at −0.5 and ±0.4 breaths per minute, respectively.

In total across both analyses, there were 1680 min of recordings from 32 participants. However, 278 min were excluded as there was no RR available from reference counts, and of the remaining 1402 min, 41 min were excluded with no RR from the Albus system. Thus, when a RR was available from the reference count, the Albus system reported a RR result 97.7% (SD = 4.6) of the time. When accuracy was assessed for all compared RR readings in the study (2694 readings from 1347 min), mean participant accuracy was 98.1% (SD = 2.7) and mean absolute error (SD) was 0.6 (0.2) breaths per minute.

## 4. Discussion

### 4.1. Clinical Relevance and Principal Findings

Albus Home is a novel nocturnal respiratory monitoring system that for the first time integrates multiple contactless sensors for automated monitoring for as long as required without adding patient burden. In this work, we have evaluated the accuracy of the RR monitoring aspect of this multi-metric system within participants’ usual home sleeping conditions and environments, which is a validation of performance in the context of real-world deployment.

Nocturnal symptoms and signs are clinically important across several cardio-respiratory conditions, such as asthma, chronic obstructive pulmonary disorder (COPD), heart failure, and cystic fibrosis [17,18,19,20], and are included in clinical guidelines for assessment of severity [21] as well as patient-reported outcome measures that are used widely in healthcare and as clinical trial endpoints [22,23]. However, nocturnal symptoms are frequently underperceived and underreported [24] by subjective tools, and in the case of RR, measuring during sleep is usually performed sporadically by manual counting in hospitalised patients.

RR is a clinically valuable vital sign [3]. Changes in RR are one of the earliest signs of deteriorations across several conditions and is an essential part of patient assessment and is included as a core part of inpatient Early Warning System scores worldwide [1,25,26]. Moreover, nocturnal RR has been shown to be predictive of deteriorations and mortality [27,28]. In the hospital setting, outside of intensive care settings, RR is measured by a visual observation of the thorax by a healthcare professional, which is limited to few sporadic measurements due to the manual, human-counted nature. Moreover, studies have found a substantial inter-rater variability in RR measurements, even when obtained by healthcare professionals, of up to 6 breaths per minute [6,29,30].

Home RR monitoring is significantly less studied than in hospitals, yet its potential value has been highlighted by prior work. For instance, Yanez et al. [2] found that mean RR significantly increased from 15.2 to 19.1 breaths per minute during the 5 days prior to hospitalisations due to COPD exacerbations. In a Bland–Altman analysis of mean RR results from the Albus system (Figure 3B), the limits of agreement were 0.4 breaths per minute, which are much narrower than the magnitude of clinically meaningful changes reported above. In that work, the RR of patients could be monitored out of hospital as they were using home oxygen therapy through oxygen delivery devices. However, outside of exceptional groups of patients using ventilatory devices at home, regular RR monitoring was not possible without adding significant burden.

Rubio et al. [10] assessed the accuracy of five different home RR monitoring systems that utilise different modalities (impedence, photo-plethysmography, camera, accelerometer, chest-band), of which the adhesive accelerometer-based (mean difference = −2.18, limits of agreement = −8.63 to 4.27) and chest-band (mean difference = −1.60, limits of agreement = −9.99 to 6.80) devices were found to be the most accurate. These two devices were then tested by patients in waking hours for 14 days. However, several acceptability issues were reported for both devices, including some patients finding them particularly intrusive when unwell, skin problems due to the adhesive patch of the accelerometer, difficulty in fitting and wearing, and difficulties in removing the device for charging.

The bedside device presented in this work overcomes the limitations of these previous RR monitors, being fully contactless and automated with no need for calibration or charging. Moreover, the accuracy of the Albus system was significantly better than the above devices with a much lower mean difference and limits of agreement of −0.4 and ±1.2 breaths per minute, respectively. These limits are much narrower than the reported limits of the above devices as well as the variability between healthcare professionals [6,29]. Even when the definition of accuracy is defined more stringently as the percentage of Albus RR readings within ±2 breaths per minute of the gold standard, mean accuracy exceeded 98%. Moreover, high accuracy was maintained across different participant characteristics, including between adults and children, healthy volunteers and those with respiratory conditions, and single-sleepers and bed-sharing participants.

### 4.2. Strengths and Limitations

A particular strength of this validation study was that it was performed in real-world conditions in the context of how it would be used. Prior validation studies have been performed in laboratory or sleep study settings, which are highly controlled environments that do not represent the variability in use by patients at home and may not accurately represent expected performance in use [7,8]. These variables can include patients not setting up a device correctly at home, patients using different sizes of beds and bedding, and importantly, the presence of a bed-sharer, which is excluded in sleep laboratory studies. In this work, participants set up the bedside device themselves after watching a brief setup video or by videocall, and they undertook the overnight monitoring in their usual bedroom environments, including usual clothing and bedding. Some participants also shared the bed with a co-sleeper as usual, with the only specified instruction being that the device should be placed on the bedside of the target participant. Thus, the results of this work represent the performance of the system as deployed in practice with the breadth of real-life variation.

Another strength of this study was the broad range in key participant characteristics with body mass indices ranging from 13–42, and the youngest and oldest participants being aged 6 and 79 years old, respectively. Though no participants younger than 6 were recruited in this study, the fundamental principles of using doppler-radar motion sensors to capture periodic movements of the chest and abdomen are applicable for all age groups, including younger children and infants. Future work will build on this present validation by deploying and evaluating the system in such groups, thereby opening wider potential applications, such as sudden infant death syndrome and viral-induced wheeze, a common presentation in pre-school children.

A limitation of this work is that it was outside its scope to characterise the clinical significance of any respiratory rates or patterns measured, though the value of both daytime and nocturnal RR has been previously demonstrated [2,3,27]. In particular, this system is designed for nocturnal monitoring, and the relationship between nocturnal RR with that of daytime was not assessed here, though prior work in other nocturnal symptoms have shown correlations between day-time and night-time [31]. Given that in the daytime there are many other variables that could significantly affect RR, such as recent physical activity, metabolic demand, and emotional arousal [32,33], we hypothesise that nocturnal RR provides less confounded readings that can be more meaningfully compared between nights. Another limitation was that the clinical states of the participants were not characterised, in part because the study was mainly carried out remotely during the coronavirus pandemic. Correspondingly, because participants were only eligible to take part if they were healthy or stable enough to be at home for those with respiratory conditions, large extremes of RR that are observed in acutely unwell patients in hospital were not observed in this study. Regardless, meaningful variations in RR were present in this study, both between and within participants, with accuracy maintained in RR readings ranging from around 10 to 24 breaths per minute (Figure 2 and Figure 3). Future work will focus on understanding the variability of nocturnal RR and minimum clinically important differences (MCID) for clinical changes. One theoretical limitation of the Albus system is that it monitors RR through capturing the periodic movements of the thorax and abdomen, rather than direct airflow from the airways. In the vast majority of cases, these align, but in certain situations such as obstructions, the thoracic or abdominal movements may not correspond to actual airflow. In a similar vein, this study focused on reporting the performance and accuracy of RR monitoring, but not specifically the absence of respiration—for instance, the detection of apnoeas. This is expected to be feasible from the set of sensors deployed in the device, but is the remit of future work.

In monitoring device validation, it is desired to assess both intersubject and intrasubject variation, ideally encompassing repeatability and reliability across multiple nights. A limitation of this study (but one shared by precedent validation studies involving polygraphy [8,15]) is that each participant undertook one night of monitoring with both the Albus system and reference. This was in large part due to the practical limitations of the gold-standard polygraphy, which is burdensome and poorly suitable for multiple nights, especially for children, but also a study design choice where given the same resource, it was hypothesised that performance was more likely to vary between different participants, rather than the accuracy for a given participant’s night significantly changing if it was repeated for the same person. To this end, the scope of this study was to evaluate the intersubject performance of the system across a broad demographic of participants, whilst assessing intrasubject performance within periods from one night. Regardless, further work is ongoing where the Albus system is deployed in longitudinal studies with nightly monitoring over several weeks and months. Thus, future work will seek to report on the performance and reliability of the system over longitudinal data.

### 4.3. Potential Applications

The contactless yet accurate nature of the Albus system opens several potential applications across clinical care and research, with the need for effective remote monitoring solutions particularly highlighted by the COVID-19 pandemic [34,35]. In a mixed methods study of asthma patients and clinicians, a key theme expressed by both groups was a solution to be automatic and requiring minimal effort from users [36]. To this end, this automated system enables the capture of accurate serial RRs much more frequently than in clinical practice, without the inaccuracies of manual measurement [6]. Since the system collects data without needing patients to do or wear anything, this allows for reliable monitoring for several weeks and months without burdening patients. Capturing detailed nightly changes in RR and other symptoms could help delineate day-to-day variations from early warning signs of deteriorations in a much broader population than was possible before [2,10,17]. In the context of clinical studies, the plug-and-play nature of this remote system, which can be easily set-up by participants themselves, also facilitates deployment in traditional trial designs as well as for remote, siteless, or decentralised trials [37,38,39]. Finally, although this present work focused on the performance of RR monitoring, a unique benefit of this system is that multiple sensors are integrated into one device, thereby enabling many different nocturnal metrics (such as coughing, sleep metrics) and environmental information (such as air quality, volatile organic compounds) to be collected without burdening participants to use multiple different devices. The validation results of these other metrics are the subject of other and future work.

## 5. Conclusions

Albus Home is a novel contactless and automated system that integrates multiple sensors for multi-metric nocturnal monitoring. This present work showed that the RR monitoring function of the system was highly accurate across a diverse population monitored in real-world conditions. Furthermore, the rapid automated analysis of data collected from the device enables longitudinal monitoring for as long as required. In conclusion, Albus Home presents a home monitoring system that enables long-term, reliable nocturnal RR monitoring with potential applications across clinical care and research.

## Figures and Tables

**Table 1:** Summary of the validation population characteristics and results.

**Participants**	32 total (24 adults, 8 children; 18 healthy volunteers, 14 chronic respiratory conditions)
**Age (range)**	Median = 29 (6–79 years)
**Body Mass Index (range)**	Median = 22 (13–42)
**Sex**	17 females, 15 males
**Bedroom occupancy**	22 single sleepers, 10 bed/room-sharers

## Data Availability

Access to data presented in this study may be available on request from the corresponding author. The data are not publicly available because some data are of a commercially confidential or sensitive nature, with intellectual property considerations.
